# Supplementary material for: Speech errors in consecutive interpreting: Effects of language proficiency, working memory, and anxiety
Source: PLoS One. 2023 Oct 18;18(10):e0292718. doi: 10.1371/journal.pone.0292718 (PMC10584118; doi:10.1371/journal.pone.0292718)
Supplement: Attachment — Submitted filename: Review comments to the authors.docx [file pone.0292718.s001.docx]

Review comments to the authors

I believe that this paper presents a novel study examining the effects of three important factors (language proficiency, anxiety, and working memory) on errors in consecutive interpreting. In the following, I have included my suggestions on needed revisions as well as some questions to the authors, which should be addressed before the manuscript is considered for publication. If these are properly addressed, then I would recommend the manuscript for publication in PLoS ONE.

Introduction and literature review:

- The literature review should include Interpreting studies on syntactic and lexical interference (e.g., Chmiel et al. 2020; Lijewska and Chmiel 2014) and studies on error detection and production reviewed by Garcia et al. (2019).

- Is there any literature to refer to on phonological interference in order to justify the expectations the authors have made on phonological errors on page 6? Here, the authors refer to Amos and Pickering’s model of prediction in interpreting, but the same authors did not find form-based (phonological) predictions after applying their model in an experimental investigation, i.e., the assumption the model makes on interlingual phonological activation was not supported experimentally, (see Amos et al. 2022).

- When it comes to findings on working memory and interpreting performance, reviews of the interpreting studies literature show that the former has an effect on the latter in trainees but not in professionals; this should be reflected in the literature review as it is quite relevant to the present study and helps support some of the findings

- The authors say “therefore, speech errors in consecutive interpreting, compared to those in

simultaneous interpreting, are more revealing of different processing stages of target language production and the influences of different cognitive factors.” The first part of this claim on processing stages makes sense, but on cognitive factors it is not clear why speech errors would be more revealing in consecutive than simultaneous interpreting. The authors should elaborate on this as simultaneous interpreting could be considered more demanding than consecutive in terms of working memory resources.

- In the example in Chinese on page 6 it is not clear why there would be syntactic errors, the rule should be explained

Methodology:

- In Table 1, a gloss should be provided for the Chinese versions as well as an explanation in the text of the error in each example. It is not at all clear where the errors lie and why these should be considered errors at the indicated level

- In Table 2, the *p* value for ‘anxiety’ should also be in bold, I believe

- An explanation is needed of why the particular test of WM used in the study was chosen, instead of other measures which are found more commonly in studies on interpreters (e.g., listening span, etc.)

Results:

Is it possible to divide participants into groups based on their scores to look at errors within each group and then compare the groups to see whether the number and types of errors differ between groups? My thought is that this would help clarify whether the effects observed in the overall participant data would be maintained across groups as scores (even if standardized) tend to vary a lot across participants. However, if the authors think differently on this point, I would be very open to read their answer and perspective.

Discussion:

- The authors should elaborate on the following statement by explaining what they mean by ‘fuzzier’ and why a lower understanding of meaning would result in more syntactic errors: “It is possible that interpreters less proficient in the source (non-native) language may have a fuzzier understanding of the source language meaning, thus are more prone to false starts or sentence organisation in their target speech, leading to more syntactic errors.”

- On page 24 the authors say, “the use of notes as an external memory sources greatly diminish possible working memory effects on lexical and syntactic errors”. Can they corroborate this with some existing findings from the literature?

- On page 25, the authors seem to conclude that anxiety affects errors because it affects working memory functions, referring to Miyake (2000) on the latter. But can they arrive at this conclusion without exploring the relationship between anxiety and working memory in their own study. Is it possible to analyse the relationship between the two factors (and language proficiency) within the scope of this study? If not, I don’t think this conclusion should be drawn.

Experimental materials (on osf.io)

Can English translations be provided for the questions in the anxiety test?

Language use mistakes (I have not included the line numbers for all of these, but I trust that the page numbers I have provided will help the authors spot and correct the mistakes:

- page 2, “the current paper…” – a verb is missing in this sentence

- page 3 ‘intension’ – check spelling

- page 6 “instead the” – preposition is missing here

- page 7 and elsewhere, rather than “in face of”, “in front of” should be used

sentence starting “Indeed, it has been shown…” should either be revised, or some text needs to be added/is missing at the end

- page 8 line 2 “language” – check spelling

line 5 “task” should be in plural

- page 9 line 8 “better in terms of” – there is additional space here

“occurrence speech” is missing a preposition

- page 10 line 9 “such that” should be “so that”

- page 12 “according the weightings” – a preposition is missing

- page 15 line 2 “the anxiety one feels that he can do a particular task” should be revised

- page 17 Figure 1 caption “one though characters” – it is not clear what is meant here/what the error is; should be corrected

- page 22 line 6 “conceptual errors decreases” – number of verb should be changed

line 12 “these” or another word needs to be removed

Table 4 caption – ‘a’ should be removed and upward arrow should be added

- page 24 “test” should be changed to “tested”

“an external memory sources” should be corrected for number

- page 26 line 2 “more importantly” is repeated

“trainee should” – number should be changed
