# Supplementary material for: Speech errors in consecutive interpreting: Effects of language proficiency, working memory, and anxiety
Source: PLoS One. 2023 Oct 18;18(10):e0292718. doi: 10.1371/journal.pone.0292718 (PMC10584118; doi:10.1371/journal.pone.0292718)
Supplement: Attachment — Submitted filename: PONE Reviews R1 responses.docx [file pone.0292718.s002.docx]

**Responses to reviews**

We would like to first express our sincere apologies for the extended duration of the revision process. This was largely due to the first author's maternity leave, which lasted until November 2022.

We have now thoroughly revised the manuscript, taking into account the insightful comments provided by the reviewers. Their careful and constructive feedback was indeed invaluable, and we extend our deepest gratitude for their time and effort.

For clarity and ease of reference, we have outlined our responses and detailed the revisions made to the manuscript, corresponding to each point raised by the reviewers. These are highlighted below: for each reviewer's comment (displayed in black), you will find our respective response and the changes made (indicated in blue). Additionally, please note that all substantial content revisions within the manuscript itself have also been marked in blue in the manuscript with track changes for your convenience.

**Mark Feng Teng**

Reviewer #1: This article is well-written. I just have some concerns on the rationale of using the the paradigm as WM test. It seems this test is highly dependent on language learners' proficiency level. I am quite surprised of not seeing the correlation results among the examined variables but directly the relative significance of each variable. The discussion section did not consider conceptual errors, syntactic errors, and phonological errors.

We sincerely appreciate the reviewer's thoughtful comments. In response to the concerns raised, we have now included a table displaying correlations among the examined variables, which can be found as Table 3 (p.).

Regarding the types of errors, we acknowledge that our previous discussion may have been dispersed throughout the document and could have been made clearer. While we understand the reviewer's perspective, we believe that a separate section discussing different error types might lead to redundancy. Therefore, instead of having a new section, we have now expanded our discussion of conceptual errors, syntactic errors, and phonological errors within the context of each cognitive factor, striving to clarify our position and findings. Thank you for bringing these points to our attention.

**Reviewer #2**: The study is novel and generally of interest for the field of interpreting studies. The empirical part of the study is quite neat, and the analysis is reliable and clearly described.

My major concern is mainly due to the cumulative effect of quite a number of minor concerns. After reading the paper, it was not 100% clear to me what the theoretical motivation of the study was, or what its implications were. Certainly, the discussion section and the motivation section did not tie in well together, and at times they did not tie in clearly with the results. I believe that before publication, the authors need to clearly motivate the study, define the terms used in the study, and then relate the discussion section back to the motivation section of the study.

There are also a number of places where flimsy claims are made that are either not logical within the paper, or else are not backed up with any citations.

We are grateful to the reviewer for acknowledging the novelty and reliability of our study, and for providing such constructive feedback. Your comments have been invaluable in guiding our revisions. Below, we address each of the concerns raised and detail the adjustments we've made in response.

Data availability

Appendices 1 and 3 are mentioned but were not in the version I received. Also, Appendix 2 is not mentioned so the numbering appears to be incorrect.

We apologize for the confusion. We have now included all the appendices on OSF.

Reference missing for Spielberger (1983). I did not check all the references – I tried to check this one since I could not find the anxiety scale on the OSF page. Anxiety scale is not on the OSF page.

Again apologies for the missing information. The reference should be Spielberger et al. (1983) and has now been added to the reference list. The anxiety test has now been added on the OSF page.

Inaccessibility of parts of the paper for non-Chinese readers.

Some information was not accessible for me as a non-Chinese speaker. There are various examples provided throughout the text in Chinese only. An English translation (word-by-word if necessary) should be provided for all of these examples.
I was also unable to understand any of the examples of types of error in Table 1

We have now added literal translation of the Chinese text and also explanations of the speech errors.

Use of terms
Cognitive resources vs. cognitive processes are not defined, working memory is not defined, anxiety is not defined, language proficiency is not clearly related to proficiency in the second language (which is in fact what this paper investigates).

We appreciate the reviewer highlighting these points. To address the concern, we have added clear definitions of the pertinent terms, such as language proficiency (p.7), working memory (p.8), and anxiety (p.8), in the manuscript. To alleviate any potential confusion, we have also decided to replace the terms 'cognitive resources' or 'cognitive factors' with 'cognitive functions.' These cognitive functions refer explicitly to language proficiency, working memory, and anxiety in our revised manuscript.

The aims of the study
The aims of the study do not always match either the empirical part of the study or the literature review. For instance:

p3: the claim is made that the research aims to shed light on language production and speech monitoring mechanisms. However, I do not find reference to this again and it is not clear how this could be done via the study described in the paper.

Thank you for pointing out this inconsistency. We have revised the introduction to better align with the study's focus and findings. The initial reference to implications for language production and speech monitoring mechanisms has been removed from the introductory paragraph. While we did, in the original submission, touch upon these aspects in the discussion section, we understand and agree that they were not central to the study's primary motivations.

In general: none of the aims of the study would involve assessing interpreting performance. This is not mentioned in either the title, abstract, motivation or conclusions of the study, and so it is surprising when on p11, we learn that performance was assessed.

In response to your comment, we have revised our manuscript to remove the element of interpreting performance from the analyses. Please note that the relationship between interpreting performance and individual differences is now the subject of a separate publication by the first author (Zhao, 2022, *Frontiers in Psychology*).

There is little motivation for why the authors hypothesized that errors at different levels would relate differently to any of the mentioned “cognitive factors”.

Thank you for this observation. While we initially based our study on existing literature which discussed how individual differences in language proficiency, working memory, and anxiety affect interpreting, we acknowledge that there hasn't been an in-depth investigation into how these individual differences relate to speech errors at different levels. As stated in our original submission, our research represents a pioneering exploratory study in this area. Therefore, while we anticipated that these individual differences would influence error occurrence, we did not propose specific hypotheses regarding the direction of these effects due to the exploratory nature of our research and as a result we did not provide motivation.

Literature review
P6: Do the sources cited (Galantucci, Fowler & Turvey; Liberman & Mattingly) mention interpreters specifically or do the authors of the paper point out that this thus extends to interpreters listening in the source language? This should be made clear.

We appreciate the request for clarification. The studies cited (Galantucci, Fowler & Turvey; Liberman & Mattingly) did not specifically examine interpreters. In response to another comment, we have now removed the section containing these references from our manuscript.

P3 and p4 – Logic: page 3 says that interpreters and speakers go through the same conversion stages (e.g., conceptualizing a message, the meaning they wish to convey) whereas p5 says that this is a difference between interpreting and speaking. This should be clarified or rephrased on page 3.

We have now removed, from p.3, the phrase “also in interpreting” to avoid confusion.

P7: Title of section “interpreting and cognitive resources” does not match content of section, which discusses the role of language proficiency, working memory, anxiety in interpreting and then the influence of these factors on speech errors.

We have now revised the section heading as “Interpreting and individual differences in cognitive functions” to better reflect the content. We thank the reviewer for this observation.

NB: The sources cited in this section generally refer to simultaneous interpreting while the study is on consecutive interpreting (e.g., Gile 2009, Gerver 1974). Would it be possible to include more sources on consecutive interpreting?

While we recognize that the majority of sources referenced pertain to simultaneous interpreting, the limited research specifically focused on the cognitive effects in consecutive interpreting necessitated this broader approach. Nevertheless, we understand the reviewer's point and have now supplemented our citations with additional studies specifically examining the cognitive effects on consecutive interpreting, such as Zhao, 2022. We believe this strengthens the connection between our work and its grounding in the literature.

P7 – citations required for claims “all of these cognitive factors have been examined in interpreting research” and “due to their close relevance to interpreting performance”.

In response to your comment, we have added the relevant citation (Zhao, 2022) to support the aforementioned claims. Please note that these statements serve as an overview, and we provide more detailed explanations, supported by additional references, in the subsequent paragraphs of our manuscript.

P8 – citation for the claim “such anxiety can become one of the major obstacles in the early stages of interpreting training.”

We have now added Zhao (2022) as a supporting reference for this statement.

P8/9: There is a bit of a mix in the last paragraph of page 8 and then moving onto page 9 between the impact of different cognitive factors on speech production in interpreting and in monolingual production. In general, there is not much evidence presented here for what is ultimately the main focus of the study.

We appreciate this point and have made revisions accordingly. A pertinent study (Zhao, 2022) examining the effects of language proficiency, working memory, and anxiety on interpreting performance and (dis)fluency in interpreting has now been included in our literature review. This added reference should strengthen our rationale for exploring the cognitive impacts on speech errors in the current study.

P9: Sentence unclear “better in terms of interpreting disfluency” – presumably the authors mean that interpreters with higher WM produce more fluent interpretations?

We have now revised this sentence. Thanks.

P9: first language appears to be equivalent to source language and non-native language, and second language is equivalent to target language or native language, which does not correspond to usual use of L1, L2 etc. Then on p10, first language once again means native language (as I think it does in the rest of the paper too).

We thank the reviewer for spotting the inconsistencies of terms. We have now used second language (L2) and first language (L1) throughout the manuscript.

P10: I do not follow the logic here. The authors previously introduced production errors in interpreting as being different from those in “normal” production because they are directly related to source language comprehension (and this is also why language proficiency in the source language is considered). Now the authors claim that consecutive interpreting is more akin to spontaneous language production and therefore more revealing of different processing stages in target language production. But this goes against the logic put forward previously. I also do not follow why speech errors in consecutive interpreting would be more revealing of the influences of different cognitive factors than speech errors in simultaneous interpreting.

We regret the confusion generated by our previous explanations. What we intended to convey was that in interpreting, CONCEPTUAL errors - though not all errors - are directly tied to the comprehension of the source language. We suggest that consecutive interpreting is a more suitable task for exploring how individual differences in cognitive functions influence the occurrence of speech errors. This is because simultaneous interpreting involves simultaneous production of the target language and comprehension of the source language. This simultaneous process could introduce numerous speech errors that might be considered "noise" in our investigation of cognitive effects on speech errors. For instance, many errors in simultaneous interpreting could simply be attributed to restarts or corrections of temporary misunderstandings of a source language sentence. To clarify these points, we have now expanded our explanation in the appropriate section (p.10).

P10 – Logic: “The research will have important pedagogical implications” because participants are student interpreters – a study that uses students as participants does not automatically have pedagogical implications.

We have now added the following justification for the pedagogical implications of the study (pp.10-11).

“Finally, we chose to focus on student interpreters, as the results of this research could have important pedagogical implications for interpreting training. The findings could guide interpreting trainers to focus on aspects of cognitive functions that tend to result in speech errors, such as emphasizing public speaking training to reduce anxiety.”

Methods
P11 – It would be helpful to state that SCIC is the European Commission’s interpretation service. It is unclear what the scale described is. The source of the original scale (Geneva University/SCIC) should be cited. If the scale only includes Accuracy/Completeness, Target Language Expression and Proper Delivery and Interpreting Strategies and Manner, then it appears to be underspecified. How were the percentages decided upon?
As we have now removed interpreting performance, this comment is no longer relevant (though we appreciate the reviewer for raising this point).

P12: Under Transcription and error coding – it is unclear how errors would arrive at a cognitive process. What does this mean?

We have now rephrased this statement as the following (p.12).

“As mentioned in the introduction, errors were categorized based on their linguistic nature—conceptual, lexical, syntactic, or phonological errors. Table 1 displays examples of each error type.”

P13: English language test. Had participants already completed this test or did they complete this test as part of their studies or as part of this study in particular?

As part of the experiment. We have now clarified this (p.14). Thank you for pointing out this ambiguity.

The text should make it clear that the anxiety scale used is a combination of Zhang and Schwarzer’s self-efficacy test and Spielberger’s trait anxiety test (I understand this to be the case).

We have followed this suggestion in our revision.

Results/Discussion
The analysis should state what percentage of errors were conceptual, what percentage were syntactic etc.

Such figures are available in Table 2 (p.16) in the original submission.

P16. Presumably it should read that total error rate increased as a function of anxiety level?

We apologize for the mistake, which is now corrected.

P18: Conceptual errors arose due to misunderstanding: given that the authors previously show that conceptual errors also occur during “normal” speaking, surely it is not now possible to conclude that all conceptual errors in consecutive interpreting are the result of misunderstanding (see also my comment re. p10 and the authors motivation for their decision to study consecutive interpreting).

We agree with the reviewer's point that conceptual errors can indeed occur during normal language production. However, in the context of interpreting, the interpreter's role is not to independently conceptualize, but rather to relay messages from the source language to the target language. This process involves comprehending a source language utterance and translating its meaning into the target language. As such, in our study, we defined conceptual errors based on a comparison between the target language meaning and the source language meaning. Thus, conceptual errors, as defined in our paper, arise from misunderstandings of the source language. To ensure this point is explicit, we have made clarifications in the relevant section (p.17).

P18: As previously mentioned “language proficiency” needs to be further specified. Here it is particularly relevant, because the authors now include syntactic, lexical and phonological errors and how they relate to language proficiency – but are assessing these errors in the Chinese output after having tested language proficiency in English and not in Chinese.

We appreciate your attention to the details. As mentioned earlier, we have now provided a precise definition of language proficiency as proficiency in a second language (L2) (see p.7, p.14). The debate over whether individuals differ significantly in their proficiency in their native/first language is indeed complex, with additional considerations like individual eloquence potentially impacting speech errors. However, we feel that this particular issue extends beyond the scope of our current paper.

Discussion
Discussion p23. “More errors in understanding the conveyed message lead to poorer interpreting performance” – previously in the results, evidence of a negative correlation between error rate and performance was established, so it seems odd to conclude here that there was a cause/effect.
Also, here, the authors conflate conceptual production errors and errors in understanding. I do not believe that comprehension can be directly measured through production in this way.

Your observation regarding the causal relationship between error rate and performance is noteworthy. However, in the revised manuscript, this issue no longer applies as we have removed the element of interpreting performance from our study. As for the linkage of conceptual errors to source language comprehension failures, please refer to our previous response for an explanation.

Discussion p24, first paragraph
It seems unusual to add in new citations in the discussion section, rather than referring back to the motivation section of the study. Perhaps this part of the discussion would have been better placed in the motivation section.

We appreciate your viewpoint on the placement of new citations within the discussion section. However, our perspective is that the discussion should not only reflect the points raised in the introduction but should also extend the discussion further, potentially introducing new literature. Especially in the case of an exploratory study like ours, where specific hypotheses regarding cognitive effects were not predefined, the emergence of new discussions and references can be instrumental in explaining the observed results.

It is unclear how the results suggest that working memory affected interpreting performance via conceptual understanding, as language proficiency did. The results presented provide an overall rating for interpreting performance based on a scale comprising conceptual, stylistic and presentation elements but do not break down the interpreting performance according to the scale.

As previously mentioned, the component of interpreting performance has now been removed from our study. However, to address your comment, it's important to note that we defined conceptual errors in interpreting as indicative of issues in source language comprehension. Hence, individual differences that influence the occurrence of conceptual errors are likely to exert their effects during the comprehension of the source language.

P24: Discussion on why working memory and language proficiency only affect conceptual understanding – the two possible accounts here only mention working memory but do not offer any explanation re. language proficiency.

We appreciate the reviewer's observation on this point. There was indeed an oversight on our part, and we should have specified that the two potential explanations were intended for the effects of working memory, not language proficiency. We have now revised the text on page 24 to reflect this accurately.

“An intriguing question arises as to why working memory, unlike language proficiency, only impacts conceptual understanding of the source language without affecting error occurrence at other linguistic levels. We propose two potential explanations, both of which require empirical verification.”

An interesting question is then why working memory, unlike language proficiency, only affects conceptual understanding of the source language but do not affect error occurrence at other linguistic levels.”

It is unclear for me why syntactic and phonological information would be “interfering” with understanding of the source language?

We appreciate the question, and we can understand the need for further clarification. The term "interfering" is used here in the context of cross-language priming, where syntactic and phonological aspects of one language might influence the processing of the other language. To better explain this, we have now incorporated additional information in the revised text on page 24 (see also below).

“(e.g., source language comprehension might interfere with target language production via cross-language structural or phonological priming; e.g., Cai et al., 2011; Jouravlev et al., 2014).”

P24: why are we now discussing error repairs when the authors previously state that error repairs are not addressed.

We apologize for the confusion. This was a mistake, which we have now corrected by removing the discussion of repairs.

P26: Conclusion? It would be helpful to conclude summing up very briefly what the study has shown.

We have now provided a conclusion section.


Style and grammar in the paper
The reader can generally always understand the meaning of the text, but there are a number of errors in English that need to be addressed. Below I have noted the errors over the first six pages but note that there are errors throughout.

Abstract, line 12, word missing: Lexical ___ increased as a function of the interpreter’s tendence of anxiety.
Thanks. Corrected.

First sentence unclear – “interpreter relays a message in the source in the target language”.
Thanks. Corrected.

Line 5, word missing: “the current paper __ whether and how”
Thanks. Corrected.

P3: Under language production. “In speaking (and also interpreting) the speaker converts a pre-verbal message...” This should perhaps be clarified as interpreting involves both speaking and listening, so perhaps should say e.g., during the production phase of interpreting
We have now removed “(and also interpreting)” in response to an earlier reviewer point.

P4, line 7: “speakers may say “the star” to refer to a small square without realizing there is a bigger star” – does not appear to make sense, please check.
Thanks. Corrected.

P4, line 15-16 (e.g., children aged 5 than 7) should read e.g., for children aged five compared to children aged seven.
Thanks. Corrected.

P5, under Speech errors in interpreting – “Interpreting largely goes through the same cognitive routines as in regular language production” – rephrase (interpreting is a process and cannot go through a cognitive routine).
Thanks. Corrected.

P5, turn of phrase “Unlike in regular language production (change to unlike regular language production).
Thanks. Corrected.

P6: Example given seems odd “the reading of the electricity” is more usually called the “meter reading” in English.

Thanks. Corrected.

**Ena Hodzik**

Reviewer #3: I have included an attachment with comments to the authors. I believe that this manuscript presents a novel study examining the effects of three important factors (language proficiency, anxiety, and working memory) on errors in consecutive interpreting. In the attached document, I have included my suggestions on needed revisions as well as some questions to the authors. If these are properly addressed, then I would recommend the manuscript for publication in your journal.

Thank you for acknowledging the novelty of our study and for recognizing the significance of the factors under examination. We sincerely appreciate your feedback and the effort you put into reviewing our work.

Review comments to the authors

I believe that this paper presents a novel study examining the effects of three important factors (language proficiency, anxiety, and working memory) on errors in consecutive interpreting. In the following, I have included my suggestions on needed revisions as well as some questions to the authors, which should be addressed before the manuscript is considered for publication. If these are properly addressed, then I would recommend the manuscript for publication in PLoS ONE.

Introduction and literature review:

- The literature review should include Interpreting studies on syntactic and lexical interference (e.g., Chmiel et al. 2020; Lijewska and Chmiel 2014) and studies on error detection and production reviewed by Garcia et al. (2019).

Thank you for directing our attention towards the pertinent studies by Chmiel et al. We have now incorporated these references into the literature review (see p.6).

Regarding the study by Garcia et al. (2019), we were unable to locate the precise reference as the details provided were not complete. We conducted a thorough search and the most relevant study we found was the following.

Santilli, M., Vilas, M. G., Mikulan, E., Caro, M. M., Muñoz, E., Sedeño, L., ... & García, A. M. (2019). Bilingual memory, to the extreme: Lexical processing in simultaneous interpreters. Bilingualism: Language and Cognition, 22(2), 331-348.

However, it didn't seem to align closely with our research focus, so we have refrained from including it. If there are additional details that could help us identify the specific study you are referring to, we would be more than happy to revisit it.

- Is there any literature to refer to on phonological interference in order to justify the expectations the authors have made on phonological errors on page 6? Here, the authors refer to Amos and Pickering’s model of prediction in interpreting, but the same authors did not find form-based (phonological) predictions after applying their model in an experimental investigation, i.e., the assumption the model makes on interlingual phonological activation was not supported experimentally, (see Amos et al. 2022).

We appreciate your insightful comments. Indeed, in light of the results reported in Amos et al. (2022), we have reconsidered our reliance on the covert simulation review for justifying our hypotheses concerning phonological errors. As a result, we have restructured our argument, now removing our discussion of covert simulation and prediction and instead using the phenomenon of cross-language phonological priming as the basis for suggesting that phonological errors may be prompted by interference from the source language (see revised section on p.6).

- When it comes to findings on working memory and interpreting performance, reviews of the interpreting studies literature show that the former has an effect on the latter in trainees but not in professionals; this should be reflected in the literature review as it is quite relevant to the present study and helps support some of the findings

We appreciate your perspective on the role of working memory in interpreting performance among trainees and professionals. However, without a specific reference supporting the claim, it's challenging for us to incorporate it into our literature review, particularly given the numerous studies we have already cited demonstrating the impact of working memory on interpreting among professionals. Moreover, a recent review on working memory and interpreting we have examined didn't contain a similar assertion. Therefore, while we don't dispute the potential validity of the claim, given the existing evidence to the contrary and its contentious nature, we have decided to refrain from incorporating it into our manuscript.

- The authors say “therefore, speech errors in consecutive interpreting, compared to those in

simultaneous interpreting, are more revealing of different processing stages of target language production and the influences of different cognitive factors.” The first part of this claim on processing stages makes sense, but on cognitive factors it is not clear why speech errors would be more revealing in consecutive than simultaneous interpreting. The authors should elaborate on this as simultaneous interpreting could be considered more demanding than consecutive in terms of working memory resources.

We thank the reviewer for raising this point, which was also raised by Reviewer #2 and was responded to above. We repeat the response below.

We regret the confusion generated by our previous explanations. What we intended to convey was that in interpreting, CONCEPTUAL errors - though not all errors - are directly tied to the comprehension of the source language. We suggest that consecutive interpreting is a more suitable task for exploring how individual differences in cognitive functions influence the occurrence of speech errors. This is because simultaneous interpreting involves simultaneous production of the target language and comprehension of the source language. This simultaneous process could introduce numerous speech errors that might be considered "noise" in our investigation of cognitive effects on speech errors. For instance, many errors in simultaneous interpreting could simply be attributed to restarts or corrections of temporary misunderstandings of a source language sentence. To clarify these points, we have now expanded our explanation in the appropriate section (p.10).

- In the example in Chinese on page 6 it is not clear why there would be syntactic errors, the rule should be explained

The error is the order of the locative in relation to the verb. We have now added a syntactically correct example to illustrate the error.

Methodology:

- In Table 1, a gloss should be provided for the Chinese versions as well as an explanation in the text of the error in each example. It is not at all clear where the errors lie and why these should be considered errors at the indicated level

We have now added translations of the Chinese texts and explanations of the errors there.

- In Table 2, the *p* value for ‘anxiety’ should also be in bold, I believe

Thanks for the spot. This is now no longer relevant as we have removed interpreting performance.

- An explanation is needed of why the particular test of WM used in the study was chosen, instead of other measures which are found more commonly in studies on interpreters (e.g., listening span, etc.)

We have now provided the following justification in the manuscript (p.14).

“In line with our established practice for gauging interpreters' working memory (R. Cai, Dong, & Zhao, 2015), we utilized a version of the reading span task, originally conceived by Daneman and Carpenter (1980) and subsequently refined by Mizera (2006).”

Results:

Is it possible to divide participants into groups based on their scores to look at errors within each group and then compare the groups to see whether the number and types of errors differ between groups? My thought is that this would help clarify whether the effects observed in the overall participant data would be maintained across groups as scores (even if standardized) tend to vary a lot across participants. However, if the authors think differently on this point, I would be very open to read their answer and perspective.

While dividing participants into groups based on their scores and comparing the errors within each group is indeed a possible approach, we chose not to use it in this study due to its potential limitations. One of the challenges with this methodology is the difficulty in controlling for other variables that might confound the relationship between the cognitive functions and errors. Cognitive functions such as language proficiency and working memory, for instance, tend to correlate with one another. This might mean that interpreters with higher language proficiency might also possess larger working memory spans, although our data did not specifically indicate this correlation. This potential correlation could lead to confounding effects that might distort our findings. Therefore, to avoid these complications, we opted for the approach presented in the study. We certainly value your suggestion and will consider such methods for future studies.

Discussion:

- The authors should elaborate on the following statement by explaining what they mean by ‘fuzzier’ and why a lower understanding of meaning would result in more syntactic errors: “It is possible that interpreters less proficient in the source (non-native) language may have a fuzzier understanding of the source language meaning, thus are more prone to false starts or sentence organisation in their target speech, leading to more syntactic errors.”

We have now got rid of the word fuzzier and use the word “superior”, as follows (p.23). We believe this now makes the statement clearer.

“It's plausible that interpreters with greater proficiency in the L2 source language possess a superior understanding of the source language meaning, hence less prone to false starts or sentence reorganisation in their output, leading to fewer syntactic errors.”

- On page 24 the authors say, “the use of notes as an external memory sources greatly diminish possible working memory effects on lexical and syntactic errors”. Can they corroborate this with some existing findings from the literature?

We have now provided a reference (Dam, 2007), which suggested that note-taking can improve, among other things, interpreters’ accuracy.

- On page 25, the authors seem to conclude that anxiety affects errors because it affects working memory functions, referring to Miyake (2000) on the latter. But can they arrive at this conclusion without exploring the relationship between anxiety and working memory in their own study. Is it possible to analyse the relationship between the two factors (and language proficiency) within the scope of this study? If not, I don’t think this conclusion should be drawn.

We don’t think we might have enough data points to conduct the suggested analysis; we therefore removed the claim in question.

Experimental materials (on osf.io)

Can English translations be provided for the questions in the anxiety test?

We have now provided English translations for the test.

Language use mistakes (I have not included the line numbers for all of these, but I trust that the page numbers I have provided will help the authors spot and correct the mistakes:

- page 2, “the current paper…” – a verb is missing in this sentence

- page 3 ‘intension’ – check spelling

- page 6 “instead the” – preposition is missing here

- page 7 and elsewhere, rather than “in face of”, “in front of” should be used

sentence starting “Indeed, it has been shown…” should either be revised, or some text needs to be added/is missing at the end

- page 8 line 2 “language” – check spelling

line 5 “task” should be in plural

- page 9 line 8 “better in terms of” – there is additional space here

“occurrence speech” is missing a preposition

- page 10 line 9 “such that” should be “so that”

- page 12 “according the weightings” – a preposition is missing

- page 15 line 2 “the anxiety one feels that he can do a particular task” should be revised

- page 17 Figure 1 caption “one though characters” – it is not clear what is meant here/what the error is; should be corrected

- page 22 line 6 “conceptual errors decreases” – number of verb should be changed

line 12 “these” or another word needs to be removed

Table 4 caption – ‘a’ should be removed and upward arrow should be added

- page 24 “test” should be changed to “tested”

“an external memory sources” should be corrected for number

- page 26 line 2 “more importantly” is repeated

“trainee should” – number should be changed

We apologize for all these typos. These have now been all corrected.
